# Therapeutic targets and potential delivery systems of melatonin in osteoarthritis

**DOI:** 10.3389/fimmu.2024.1331934

**Published:** 2024-01-24

**Authors:** Zhilin Xiong, Guoxuan Peng, Jin Deng, Miao Liu, Xu Ning, Yong Zhuang, Hua Yang, Hong Sun

**Affiliations:** ^1^ Department of Orthopaedics, The Affiliated Hospital of Guizhou Medical University, Guiyang, China; ^2^ Department of Emergence Surgery, The Affiliated Hospital of Guizhou Medical University, Guiyang, China

**Keywords:** osteoarthritis, melatonin, inflammation, oxidative stress, chondrocyte death, delivery systems

## Abstract

Osteoarthritis (OA) is a highly prevalent age-related musculoskeletal disorder that typically results in chronic pain and disability. OA is a multifactorial disease, with increased oxidative stress, dysregulated inflammatory response, and impaired matrix metabolism contributing to its onset and progression. The neurohormone melatonin, primarily synthesized by the pineal gland, has emerged as a promising therapeutic agent for OA due to its potential to alleviate inflammation, oxidative stress, and chondrocyte death with minimal adverse effects. The present review provides a comprehensive summary of the current understanding regarding melatonin as a promising pharmaceutical agent for the treatment of OA, along with an exploration of various delivery systems that can be utilized for melatonin administration. These findings may provide novel therapeutic strategies and targets for inhibiting the advancement of OA.

## 1 Introduction

Osteoarthritis (OA) is a prevalent age-related irreversible musculoskeletal disorder, recognized as a primary cause of chronic pain and disability. It is characterized by persistent synovitis, progressive degradation of articular cartilage, secondary formation of osteophytes, and remodeling of subchondral bone (1–3). The pathogenesis of OA is influenced by a myriad of risk factors, encompassing age, obesity, gender, genetic predisposition, and joint injuries (4). As a refractory condition, OA can not only give rise to localized symptoms such as pain, joint deformity, and joint dysfunction but also coexist with comorbidities including diabetes, cardiac ailments, and mental health disorders, which significantly augments the likelihood of serious adverse events (5). The global prevalence of OA stands at approximately 7% of the world’s population, equating to around 500 million individuals, and the number continues to rise due to the worldwide obesity epidemic and the aging demographic (6, 7). The high incidence of adverse effects and the rapid increase in the prevalence of OA impose a substantial financial burden on society, families, and individuals, while also posing a significant threat to public health (8, 9).

The main pathological characteristics of OA include the loss of chondrocytes, degradation of the cartilage matrix, and synovitis, ultimately leading to terminal OA (10). The treatment for OA involves halting the loss of chondrocytes, promoting the production of cartilage matrix, and reducing synovitis. A wide range of therapeutic approaches have been employed for the treatment of OA, including minimally invasive surgery, conventional surgical procedures, muscle strengthening exercises, physiotherapy interventions, sodium hyaluronate injections, corticosteroids administration, and nonsteroidal anti-inflammatory drugs (NSAIDs) (11, 12). Furthermore, several emerging therapeutic strategies have demonstrated promising initial outcomes, including the transplantation of autologous chondrocytes, and the intra-articular administration of platelet-rich plasma and mesenchymal stem cells (MSCs) (13–15). Unfortunately, current therapies for individuals with OA yield unsatisfactory outcomes due to the lack of effective interventions to impede chondrocyte loss and articular cartilage deterioration (16). The investigation of novel therapeutic targets for this intricate disease is thus imperative.

The endogenous indole hormone melatonin is primarily secreted in the pineal gland, synthesized from tryptophan through a series of derivative reactions (17). The release of melatonin into the circulation of cerebrospinal fluid and bloodstream facilitates its subsequent delivery to distant organs and tissues to regulate inflammation, provide antioxidant protection, inhibit tumor growth, and promote anti-aging effects (18–21). The findings of multiple studies have demonstrated that melatonin exerts a protective effect against the development of OA through mechanisms such as inflammation reduction, elimination of excess free radicals, and promotion of matrix synthesis (22). Consequently, the potential clinical application of melatonin characterized by minimal adverse effects, holds great promise as a viable strategy for the treatment of OA. The intra-articular injection of melatonin is an optimal choice due to the absence of lymphatic and circulatory networks in hyaline cartilage. However, due to the short half-life of melatonin, it is necessary to administer injections as frequently as twice a week (23). To minimize the frequency of intra-articular injection, several delivery systems have been employed for the sustained release of melatonin. The present review provides an overview of the therapeutic advantages and delivery systems of melatonin in the progression of OA. These findings may offer a comprehensive understanding of forthcoming studies on melatonin-based treatment for OA.

## 2 The role of oxidative stress, inflammation, and chondrocyte death in OA

### 2.1 Oxidative stress in OA

The imbalance between oxidation and antioxidants leads to oxidative stress (24). Reactive oxygen species (ROS), which are byproducts generated during aerobic metabolism, are unstable and reactive molecules such as superoxide anion (O_2_^-^), hydroxyl radical (OH-), hydrogen peroxide and (H_2_O_2_). The catalysis of ROS occurs in peroxisomes and mitochondria through the action of Nitric Oxide Synthase (NOS), Xanthine Oxidase (XO), NADPH oxidases (NOXs) (25). Under physiological conditions, O_2_^-^ is the most abundant type of ROS, with the majority being generated by mitochondria. Mitochondria, known as the “powerhouse” of eukaryotic cells, convert nutrient molecules into adenosine triphosphate (ATP) through oxidative phosphorylation (26). Although the conventional consensus posits that chondrocytes derive their energy through anaerobic glycolysis in an oxygen-deprived environment, the ample oxygen supply on the surface area of articular cartilage fosters conducive conditions for aerobic respiration (27, 28). The respiratory chain, located in the inner membrane of mitochondria, is widely recognized as the primary source of ROS and generates approximately 2%–3% of O_2_^-^ as a byproduct during oxidative phosphorylation (29). Additionally, mitochondria play a crucial role in regulating the synthesis of antioxidant systems such as NADH/NAD+, NADPH/NADP+, and GSH/GSSG. In pathological conditions, however, mitochondrial homeostasis is disrupted, leading to an excessive generation of O_2_^-^. Excessive production of O_2_^-^ leads to mitochondrial dysfunction by reducing the membrane potential of mitochondria and causing damage to mitochondrial DNA (mtDNA), thereby amplifying the generation of O_2_^-^. Not only does H_2_O_2_ originate from XO during the conversion of hypoxanthine to xanthine, but it can also be generated from O_2_^-^ upon activation of superoxide dismutase (SOD). Reactive nitrogen species (RNS) encompass a group of reactive molecules derived from O_2_^-^ and NO, which are accountable for inducing nitrosative stress that contributes to cellular damage. Endothelial NOS (eNOS), neuronal NOS (nNOS), and inducible NOS (iNOS) represent three distinct isoforms of nitric oxide synthase. The production of NO is attributed to the activity of three NOSs, namely nNOS, eNOS, and iNOS. While nNOS and eNOS generate NO at a significantly low level, iNOS induced by inflammatory cytokines such as interleukin-1β (IL-1β), IL-17, and tumor necrosis factor α (TNFα) exhibits a relatively high output of NO (30, 31).

The cells possess antioxidant defense mechanisms comprising both enzymatic and non-enzymatic components to counteract the heightened production of ROS and prevent cellular dysfunction. The non-enzymatic system comprises ascorbic acid (vitamin C), α-tocopherol (vitamin E), and glutathione (GSH), while the enzymatic component consists of SOD, catalase (CAT), glutathione peroxidase (GPX), peroxiredoxins (PRXS), and NADPH ubiquinone oxidoreductase (NQO1) (32). The SODs, comprising three isoforms including cytosolic SOD (SOD1), mitochondrial SOD (SOD2), and extracellular SOD (SOD3), effectively eliminate ROS by converting O_2_^-^ to H_2_O_2_. Subsequently, the accumulated H_2_O_2_ is further converted to H_2_O through the actions of GPX, PRXS, and CATs (33–35). The presence of GSH is crucial for maintaining cellular redox potential and antioxidant defenses, as it serves as a significant reductant. GPX plays a vital role in preventing the oxidation of membrane lipids by converting H_2_O_2_ to H_2_O through the oxidation of GSH to GSSH (35). The downregulation of antioxidant system proteins, including SOD, CAT, and GPX, has been observed in both *in vivo* and *in vitro* studies of OA joints (36). When the production of ROS exceeds the scavenging capacity of the antioxidant system or the low activity of the antioxidant defense system, the cell is in a condition of oxidative stress which is characterized by an imbalance of oxidation and antioxidant state (37). The pathogenesis of numerous age-related disorders has been strongly associated with oxidative stress, which also serves as a pivotal contributor to the progression of OA (38–41).

**Figure 1:**
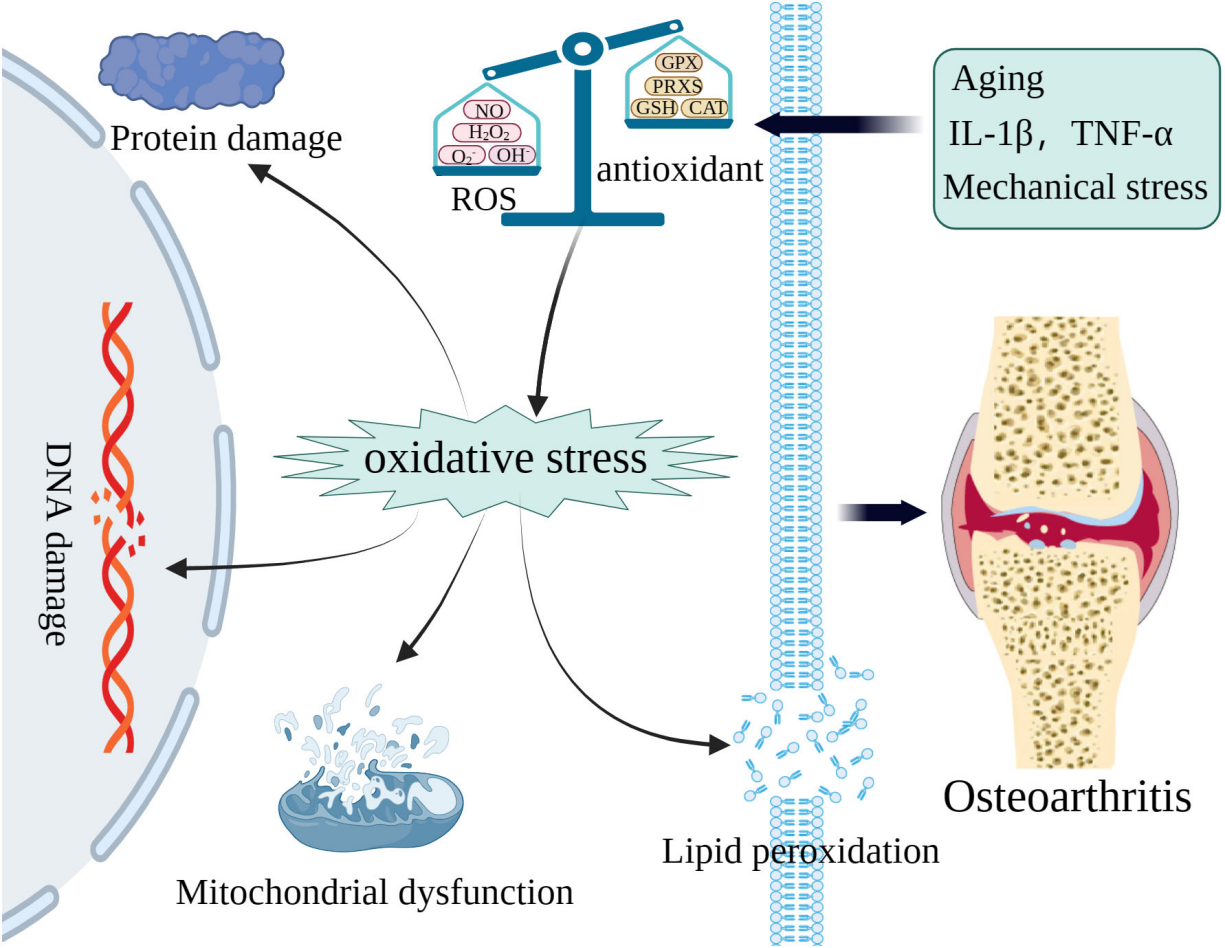
The excessive ROS caused by various pathologic processes can oxidize macromolecules including mitochondrial DNA (mtDNA), genomic, proteins, and lipids to accelerate OA.

The maintenance of cellular function and homeostasis necessitates a physiological level of ROS, however, excessive ROS induced by pathological processes can oxidize macromolecules such as mtDNA, genomic DNA, proteins, and lipids, thereby impairing essential cellular processes (**Figure 1**) (42–44). Investigations have documented that ROS-induced macromolecule compromise, including that of genomic, mtDNA, and lipids, results in synovitis worsening, extracellular matrix (ECM) degradation, and chondrocyte death, such as apoptosis and ferroptosis (45, 46). The increased level of ROS in cartilage and chondrocytes can be attributed to variations in oxygen pressure, mechanical stress, as well as the presence of inflammatory mediators such as IL-1, IL-17, and TNF-α (29, 47). The upregulation of ROS levels in the chondrocytes of individuals with OA have been demonstrated by numerous studies (45, 48). The most predominant ROS found in OA cartilages and chondrocytes are O_2_^-^ and H_2_O_2_. Excessive generation of O_2_^-^ can activate the transcription factor NF-κB, subsequently leading to elevated levels of cytokines, chemokines, and iNOS (49). Meanwhile, the cartilages and chondrocytes of individuals affected by OA also exhibit an excessive production of NO and its derivative (50). The anabolism of proteoglycans is hindered by abnormal levels of H_2_O_2_ and NO, thereby impeding the production of cartilage matrix (51). In addition, studies have demonstrated that exposure of chondrocytes to pro-oxidants such as H_2_O_2_, tert-butyl hydroperoxide (TBHP), and menadione disrupts cellular redox equilibrium and induces oxidative stress, thereby leading to increased inflammation, apoptosis, and ferroptosis (52, 53). Moreover, oxidative stress accelerates telomere shortening and impairs chondrocyte replication capacity, thereby promoting chondrocyte senescence (39). A significant contributing factor to OA is the senescence of chondrocytes, which compromises the redox balance of mitochondria and leads to an increased production of ROS, which can result in the oxidation of genomic and mtDNA (54–56). Consequently, this oxidative damage can accelerate chondrocyte senescence and impede chondrocyte proliferation (57, 58). Taken collectively, these studies demonstrate that oxidative stress induced by excessive production of ROS under various adverse conditions promotes the degradation of cartilage, hinders ECM synthesis, and induces chondrocyte senescence and death. All these effects contribute to the progression of OA. Consequently, developing therapeutic interventions targeting detrimental ROS may hold promise for OA treatment.

### 2.2 Inflammation in OA

A fundamental defensive response to an infection stimulated by microorganisms or antigens is inflammation, which is mediated by the host immune system. The short-term, regulated inflammation contributes to tissue defense and repair, whereas the long-term, aberrant inflammation leads to tissue damage and cell death. The pathophysiology of several human diseases, such as diabetes, obesity, cancer, neurological diseases, and autoimmune diseases, are significantly influenced by chronic inflammation (59). It is well established that inflamed synovium is now recognized as a prevalent indicator of OA. To maintain the proper functioning of articular cartilage, the synovium produces synovial fluid containing hyaluronic acid and lubricin. It has been observed that patients in advanced stages of OA exhibit elevated levels of chemokines and proinflammatory cytokines in their synovial fluid (60, 61). The degeneration of cartilage and the exacerbation of synovitis are both attributed to the overexpression of prostaglandins, leukotrienes, chemokines, and cytokines in the synovium (62). Generally, chondrocytes are typically situated in an anaerobic environment, which helps maintain the articular cartilage in a state of low metabolic activity and limited turnover synthesis of ECM. However, under pathological conditions, chondrocytes overproduce chemokines and cytokines that enhance the levels of collagenases and aggrecanases, thereby disrupting the delicate balance between anabolism and catabolism in articular cartilage and leading to erosion of ECM (63). Further investigations have revealed that the elevation of cytokine levels in joints plays a pivotal role in the pathogenesis of OA by regulating oxidative stress and chondrocyte death (64). Consequently, targeting anti-inflammatory strategies hold significant potential for the treatment of OA.

The three most prominently expressed cytokines in patients with OA are IL-1, IL-6, and TNF-α, which are produced by macrophages, chondrocytes, and fibroblast-like synoviocytes, which play a significant role in the degenerative process of OA (29). Other cytokines, such as IL-17, IL-18, CXCL5, RANTES, and MCP1, have also been demonstrated to serve as key regulators in the pathogenesis of OA (65, 66). Intra-articular injections of either TNF-α or IL-1 into the knee joints have been demonstrated to expedite the progression of OA, with their combined effects further exacerbating this impact (29). The expression of catabolic genes such as COX-2, IL-6, iNOS, a disintegrin and metalloproteinase with thrombospondin motifs (ADAMTSs), and matrix metalloproteases (MMPs) was found upregulated in chondrocytes stimulated with IL-1 and TNF-α, while the expression of anabolic genes including collagen II and aggrecan was downregulated (67–69). The aberrant expression of iNOS induced by inflammatory cytokines enhances the expression of NO, thereby increasing the level of IL-1β and TNF-α to aggravate inflammation through activation of the NF-κB pathway (70). It is well established that MMPs and ADAMTSs are responsible for the degradation of collagen and aggrecan, respectively (71, 72). The pro-inflammatory cytokines TNF-α and IL-1 could inhibit the function of complex I, membrane potential, and lead to mtDNA damage, therefore contributing to mitochondrial dysfunction in human chondrocytes (28). It has been reported that the production of functionally compromised respiratory chain subunits was indued by mtDNA damage and mutations, thereby increasing the levels of ROS in chondrocytes (73). The impaired mitochondrial bioenergetics and increased inflammatory response ultimately contribute to chondrocyte death (73). Treatment of chondrocytes with inflammatory cytokines such as IL-1 and TNF-α led to a significantly elevated level of IL-6 and MMP-13 (74–76). Additionally, the administration of IL-6 through intra-articular injection in mouse knee joints promoted the destruction of articular cartilage (77). Therefore, these findings indicate that inflammatory cytokines are involved in perturbing the homeostasis of articular cartilage to involve the development of OA, and the stability of the inflammatory microenvironment is responsible for determining the function of joints.

### 2.3 Chondrocyte death in OA

Cell death plays a vital role in maintaining homeostasis and the developing of the body by eliminating senescent cells and shaping tissue during embryologic development. Additionally, cell death is an aberrant pathological phenomenon triggered by detrimental stimuli such as infections and injuries (78). The sole cell type found in articular cartilage, chondrocytes, are intricately embedded within the ECM and play a vital role in maintaining ECM homeostasis by regulating anabolic and catabolic processes, as well as repairing the damaged cartilages in OA. Therefore, the loss of chondrocytes may accelerate the remodeling of ECM, leading to abnormal structure of ECM and articular cartilage degeneration, thereby potentially hastening the progression of OA. Consequently, strategies to protect from the degeneration of articular cartilage can be developed by understanding the molecular mechanism of chondrocyte death. According to the regulation of involved processes, chondrocyte death can be categorized into non-programmed and programmed forms. Autophagy, pyroptosis, ferroptosis, and necroptosis are all examples of programmed cell death (PCD), while necrosis is a form of non-programmed cell death (non-PCD) that occurs due to chemical or physical stimulation under extreme conditions (79, 80).

Autophagy is a crucial cellular process responsible for the elimination of misfolded proteins, damaged organelles, and intracellular pathogens to maintain cellular homeostasis (81–83). Autophagy can be categorized into three distinct types, including macroautophagy, microautophagy, and chaperone-mediated autophagy. Macroautophagy, commonly known as autophagy, involves the formation of bilayer membranes derived from the endoplasmic reticulum (ER) and intracellular components that encapsulate proteins and organelles, and eventually fuse with lysosomes to form autophagolysosomes (84, 85). Lysosomes contain a high concentration of hydrolytic enzymes that are capable of breaking down various substrates, including damaged macromolecules and organelles. The autophagy process consists of several consecutive phases, namely initiation, phagophore or nucleation maturation, membrane elongation, sequestering the target substrate and autophagosome formation, lysosome fusion, and substrate degradation (86–88). The autophagy process is regulated by approximately 40 autophagy-related genes, with the majority of ATG functioning in complexes to regulate autophagy through various signaling pathways (86, 89). Autophagy serves as a defense mechanism for maintaining intracellular homeostasis, operating at a basal level under normal conditions to eliminate aging-related damaged organelles and misfolded proteins (90). Autophagy can also be triggered by extreme conditions, such as external pressure, limited nutrient availability, hypoxia, and endoplasmic reticulum stress (ERS). The upregulation of autophagy-related proteins, including Unc-51-like kinase 1 (ULK1), LC3, and beclin-1, has been confirmed in human chondrocytes, however, the levels of these proteins decline in the aging population (91). The insufficient level of autophagy fails to effectively eliminate damaged organelles and macromolecules, leading to the disruption of chondrocyte homeostasis and ultimately resulting in OA (92). Therefore, the age-related decline in autophagy is a contributing factor to the deterioration of articular chondrocytes, thereby being associated with the occurrence and progression of OA.

Apoptosis, a tightly regulated mechanism of cell death, is indispensable for maintaining tissue homeostasis and ensuring the proper functioning of the human body. Morphological characteristics associated with cell apoptosis include DNA fragmentation, chromatin condensation, cell shrinkage, membrane blistering, and the formation of apoptotic bodies (93). Previous studies have shown that chondrocyte apoptosis is related to articular cartilage degradation (94). The intrinsic mitochondrial pathway and the extrinsic death receptor pathway are two well-established signaling pathways for apoptosis (79). External stimuli induce an increase in mitochondrial membrane permeability, facilitating the release of apoptotic factors such as cytochrome C and procaspases into the cytoplasm, thereby triggering activation of the mitochondrial pathway (95, 96). Under normal circumstances, damaged or depolarized mitochondria are selectively eliminated through autophagy to prevent cellular damage caused by dysfunctional mitochondria, which is commonly referred to as mitophagy (97, 98). The insufficient clearance of dysfunctional mitochondria through mitophagy leads to the release of apoptotic factors into the cytoplasm and subsequent initiation of apoptosis. This process is further exacerbated by the excessive production of ROS (99). The chondrocytes exhibited impaired autophagy and excessive apoptosis during the later stages of OA. Moreover, the essential anti-apoptotic proteins, such as Bcl2 and Bcl-XL, can suppress autophagy by binding to the key regulators of autophagy Beclin 1, thereby inhibiting the formation of the Beclin 1 complex. The apoptosis appears to be intricately linked with autophagy. The relationship between apoptosis and autophagy in chondrocytes remains incompletely understood, necessitating further investigation for confirmation.

The different forms of cell death are classified as lytic or non-lytic based on whether the cellular contents overflow upon cell death (100). Pyroptosis, also referred to as inflammatory necrosis, is a specific form of lytic cell death primarily triggered by diverse inflammasomes. These inflammasomes, such as the NLR family pyrin domain containing 3 (NLRP3), assemble in the cytosol and activate caspase to cleave gasdermins, generating membrane toxic enzymes that contribute to the formation of cell membrane perforation (101). The influx of water into the cytosol triggers a progressive swelling of cells, ultimately leading to membrane rupture. This event results in the release of cellular debris and cytokines, which not only impair neighboring cells but also exacerbate inflammation (102–104). Pyroptosis, similar to apoptosis, is a form of caspase-dependent PCD. Pyroptosis consists primarily of two pathways, including the non-canonical pathway and the canonical inflammasome pathway (105). The non-canonical inflammasome pathway is mediated by caspases 4, 5, and 11, whereas the canonical inflammasome pathway is mediated by caspase-1. Pyroptosis has been implicated in the pathogenesis of various diseases, including respiratory, circulatory, digestive, and urinary tract disorders since its original proposal in 2001 (106–109). The involvement of chondrocyte pyroptosis in the pathogenesis of OA has been experimentally validated (110). In addition to being commonly associated with OA, obesity, age, and basic calcium phosphate (BCP) also possess the ability to activate the NLRP3 inflammasome, thereby triggering chondrocyte pyroptosis (80). The expression of pyroptosis-related inflammasomes is upregulated in the synovial fluid of individuals affected by OA. Moreover, overexpression of inflammasomes enhances the levels of inflammatory factors such as IL-1β and IL-18, both contributing to chondrocyte pyroptosis and inflammatory responses (80). Additionally, the suppression of OA deterioration can be achieved by inhibiting the NLRP3 inflammasome with CY-09 (111).

Initially proposed by Stockwell’s team in 2012, ferroptosis represents a distinct form of PCD (112). In contrast to autophagy, apoptosis, and pyroptosis, ferroptosis is an iron-dependent PCD characterized by unique morphological features including mitochondrial structural disruption and accumulation of lipid peroxides (113). The distinguishing features of ferroptosis from other PCDs primarily lie in the morphological changes observed in mitochondria, such as reduction or disappearance of mitochondrial cristae, decrease in mitochondrial volume, and rupture of the outer membrane (114). Iron-ion plays a crucial role in the process of ferroptosis, as it facilitates the generation of abundant ROS through the Fenton reaction, consequently leading to the formation of lipid peroxides (115). The accumulation of lipid peroxides ultimately contributes to an increase in membrane permeability and subsequent cell membrane rupture, resulting in cell death. Under normal circumstances, the essential antioxidant defense system known as glutathione peroxidase 4 (GPX4) effectively prevents the buildup of lipid peroxides, thereby mitigating ferroptosis (53, 116). The level of iron ion in the cartilage synovial fluid of the OA group has been found to be significantly higher *in vivo*, while the level of GPX4 is lower compared to that in the normal group (53). Furthermore, ferroptosis can enhance the upregulation of MMP13 and downregulation of collagen II, thereby exacerbating ECM degradation (113). A growing body of studies has demonstrated that ferroptosis plays a significant role in the pathogenesis of OA (117, 118). The occurrence of other forms of cell death, such as cuproptosis, in addition to the previously discussed chondrocyte death, is also closely associated with the onset of OA (94, 119).

## 3 Melatonin targeting oxidative stress, inflammation, and chondrocytes death in OA

### 3.1 Melatonin

The fat-soluble indole hormone melatonin (N-acetyl-5-methoxytryptamine) was initially isolated by Aaron B. Lerner and colleagues in 1958 (120). The synthesis of melatonin in mammals primarily occurs in the pineal gland, although it is also secreted by non-pineal cells and tissues such as lymphocytes, platelets, megakaryocytes, retina, ovary, testis, liver, and skin. These extrapineal sources of melatonin function in an autocrine or paracrine manner (121, 122). The production of melatonin exhibits a distinct circadian rhythm, being synthesized predominantly during the night and suppressed during the day (123). Melatonin is biosynthesized through a complex enzymatic pathway originating from the essential amino acid tryptophan under the catalytic action of a series of enzymes (124). Melatonin is rapidly delivered to its targeted cells or organelles via the bloodstream or cerebrospinal fluid upon production (125). Once integrated with the target, melatonin exerts a diverse range of physiological effects through both receptor-dependent and receptor-independent pathways (126, 127). The melatonin receptors 1 (MT1) and melatonin receptor 2 (MT2) are G-protein-coupled receptors that are localized on both the mitochondria and the cell membrane. In addition, there is a cytosolic melatonin receptor 3 (MT3) found in several species but absent in humans. Furthermore, nuclear binding receptors such as retinoid acid-related orphan receptors (RORs)/RZR also function as receptors that melatonin targets (128, 129). The production of MT1 and MT2, which respectively regulate rapid eye movement sleep and non-rapid eye movement sleep, can be synchronized by melatonin in physiological sleep to regulate circadian rhythms (130). In addition to targeting MT1 and MT2 receptors for circadian rhythm modulation, melatonin also interacts with nuclear receptors such as RORs to modulate the circadian rhythm (131). Alongside regulating circadian rhythms, the binding of melatonin to MT1 and MT2 receptors enhances the expression of silent information regulator 1 (SIRT1) while inhibiting the phosphorylation of p38 and JNK MAPKs, thereby facilitating cell survival (132). Moreover, melatonin acts as an effective scavenger of free radicals by activating antioxidant enzymes and reducing the damaged cellular macromolecules and organelles through a receptor-independent pathway (133–135). The latest research has demonstrated that melatonin exerts a mitigating effect on inflammation, oxidative stress, and chondrocyte death in order to prevent cartilage destruction and further deterioration of OA, and the effects of melatonin on animals are listed in **Table 1**.

**Table 1:** A list of reports studying the effect of melatonin on animal.

Animal	Model	Treatment	Effect	Reference
Twelve-week-old male Sprague-Dawley rats	DMM	Intraarticular injection 100 μL MT (10 mg/mL) for one month	Prevents Cartilage Degrada -tion in DMM-induced OA	Zhou et al. (136)
Seven-week-old male Sprague-Dawley rats	DMM	Intraarticular injection 100 μL MT (10 mg/mL) once a week for twelve weeks.	Recharges of chondrocyte mitochondria to protect ca- rtilage matrix homeostasis	Zhang et al. (137)
Sprague-Dawley rats (weight 210±20 g)	ACLT	Intraarticular injection 10 mg/kg or 20 mg/kg MT (10 mg/mL) once a wee for one month.	Inhibites matrix metallopr- oteinasesa in a concentration -dependent manner	Zhao et al. (138)
Eight-week-old male Sprague–Dawley rats	Intraarticular injection collagenase	Subcutaneous injection 10 mg/kg MT (10 mg/mL) twice daily for one month	Downregulates the levels of MMP-13 and upregulates the expression of COL2A1.	Hong et al. (139)
Five-month-old male Sprague–Dawley rats	ACLT	Intraperitoneal injection 20 mg/kg or 60 mg/kg MT (10 mg/mL) once daily for six weeks	Abolishes proinflammatory factor expression in a conc- entration -dependent manner	Liu et al. (140)
Nine-week-old male C57BL/6J mice	DMM	Intraarticular injection 10 μL MT (10 mg/mL) twice a week for one month.	Prevents Cartilage Degradat -ion in DMM-induced OA	Zhang et al. (141)
Six–seven month-old female New Zealand white rabbits	ACLT	Intraarticular injection 20 mg/kg MT weekly for one month.	Anti-inflammatory effects to ameliorate OA	Lim et al. (142)
Eight-week-old female C57BL/6 mice	ACLT	Intraarticular injection 10 µL MT (50 mM) twice a week for eight weeks	Exerts protective effect on chondrocytes against inflammatory damage	Liang et al. (143)
Eight-week-old male Sprague-Dawley rats	DMM	Intraperitoneal injection (15mg/k- g ) or (30 mg/kg) MT (10 mg/mL) every other day for eight weeks.	Anti-Apoptosis and Autop- hagy effects in a concentra- tion-dependent manner	Chen et al. (144)
Ten–twelve week-old male C57/BL mice	ACLT	Intraperitoneal injection (50 mg/k- g) or (150 mg/kg) MT (10 mg/mL) once a day for eight weeks	Attenuates mouse chondroc- yte apoptosis in a concen- tration-dependent manner	Qin et al. (145)

MT, melatonin; OA, osteoarthritis; DMM, destabilization of the medial meniscus; ACLT, anterior cruciate ligament transection.

### 3.2 Melatonin as an inhibitor of oxidative stress

**Figure 2:**
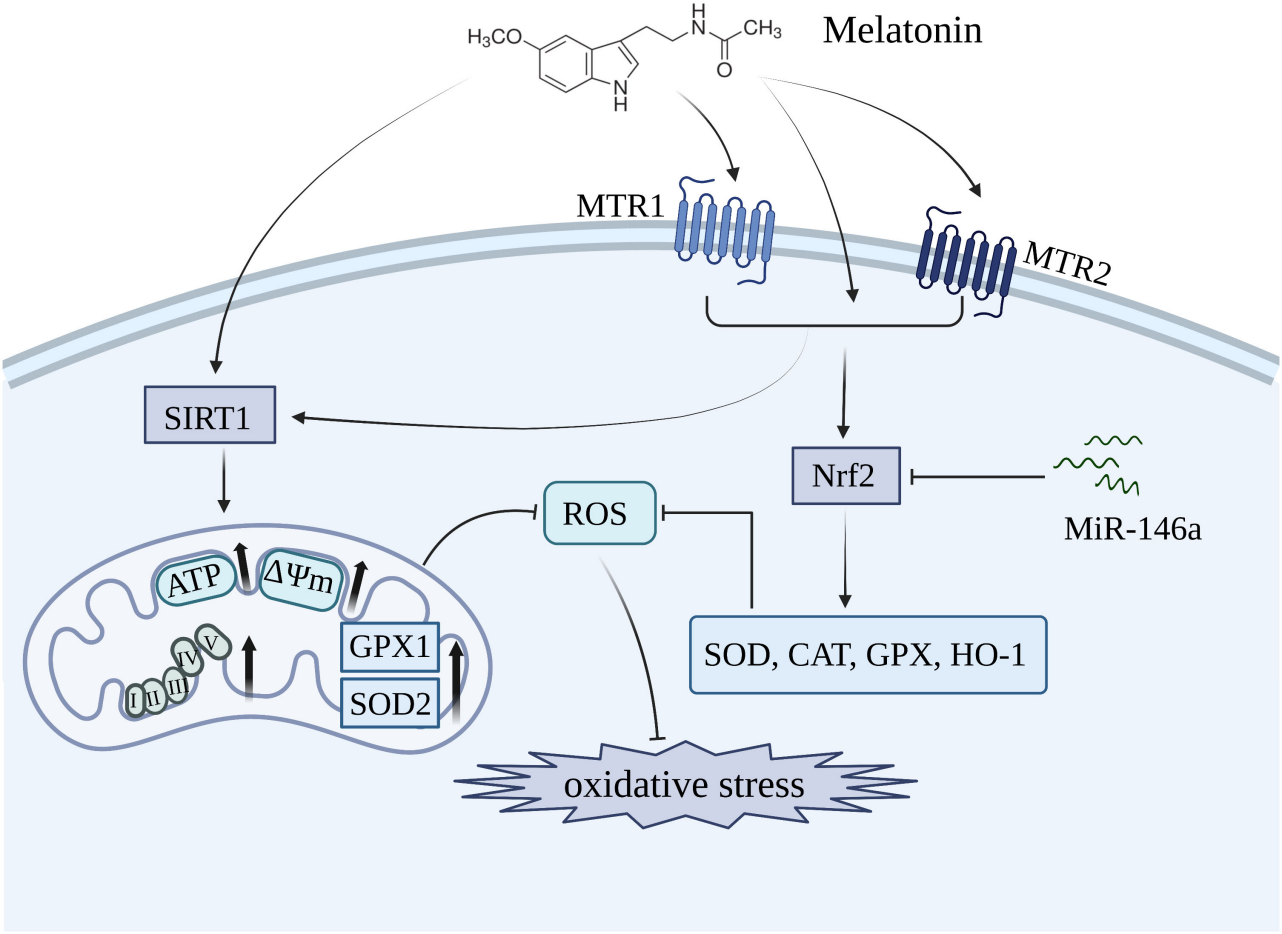
Melatonin inhibits oxidative stress in OA by restoring mitochondrial homeostasis and enhancing the level of antioxidant enzymes including SOD, CAT, and GPX. MTR1, melatonin receptor 1; MTR2, melatonin receptor 2; SIRT1, silent information regulator 1; Nrf2, nuclear factor-erythroid 2-related factor 2.

The hydrophilic and lipophilic properties of melatonin enable it to traverse all biological barriers, exerting an antioxidative impact on the cytosol, mitochondria, and cellular membrane (146, 147). Melatonin not only directly scavenges free radicals, but also enhances the activity of antioxidant enzymes such as SOD, CAT, and GPX to effectively inhibit oxidative stress (135, 148–150) (**Figure 2**). The nuclear factor-erythroid 2-related factor 2 (Nrf2) functions as a crucial transcription factor for antioxidant defense. Melatonin acts as an effective antioxidant, regulating the homeostasis of the cartilage matrix through the Nrf2 signaling pathway. This is evidenced by the increased expression of Nrf2 in melatonin-treated chondrocytes, which led to a reduction of intracellular ROS levels and a significant elevation in the expression of SOD1, SOD2, CAT, and HO-1 (136). The expression of Nrf2 and antioxidant enzymes could be significantly inhibited by miR-146a, which was markedly elevated in OA chondrocyte. Moreover, overexpression of miR-146a reduced the level of Nrf2, thereby diminishing the protective effects of melatonin in articular cartilage of rats (136).

Mitochondria, the primary producers of ROS, serve as the key target organelles for melatonin in inhibiting oxidative damage. An *in vitro* showed that melatonin treatment restored mitochondrial homeostasis in OA chondrocytes by upregulating the expression of ATP, mtDNA, and respiratory chain factors such as CoxIV2, Sdha, Nd4, and Atp5a, thereby leading to a reduction in mitochondrial ROS levels and promotes an antioxidative effect (137) (**Figure 2**). The antioxidative benefits of melatonin, however, are compromised in mitochondrial homeostasis when the expression of SOD2 is inhibited, suggesting that SOD2 plays an essential role as a downstream component in mediating the protective effects of melatonin. Additionally, SIRT1, a histone deacetylase enzyme involved in nicotinamide adenine dinucleotide (NAD+) metabolism, is crucial for maintaining the activities of antioxidative enzymes (151, 152). Patients with OA who exhibited lower levels of SIRT1 showed an accelerated deterioration of articular cartilage, thereby suggesting that SIRT1 plays a protective role in the development of OA (153). The administration of melatonin significantly enhanced the expression of SIRT1, thereby promoting SOD2 activity and expression through its involvement in histone deacetylation. In contrast, the inhibition of SIRT1 significantly diminished the protective effects of melatonin, suggesting that melatonin plays a crucial role in maintaining mitochondrial function to suppress oxidative stress by modulating the level of SIRT1 in OA progression (137). The effects of melatonin on OA through SIRT1 are listed in **Table 2**.

**Table 2:** The effects of melatonin on OA via SIRT1 .

Reference	Signaling pathway	Effect
Lim et al. (142)	SIRT1/NF-κB	Modulate the anti-inflammatory effects
Guo et al. (154)	SIRT1**/**NAMPT and NFAT5	Attenuate MMP-3 and MMP-13 production
Zhang et al. (137)	SIRT1/SOD2	Regain the chondrocyte mitochondrial function
Zhao et al. (155)	SIRT1/NF-κB	Prevent chondrocyte matrix degradation
Qin et al. (145)	SIRT1/IRE1α- XBP1- CHOP	Eliminate chondrocyte apoptosis

### 3.3 Melatonin as an inhibitor of inflammation

**Figure 3:**
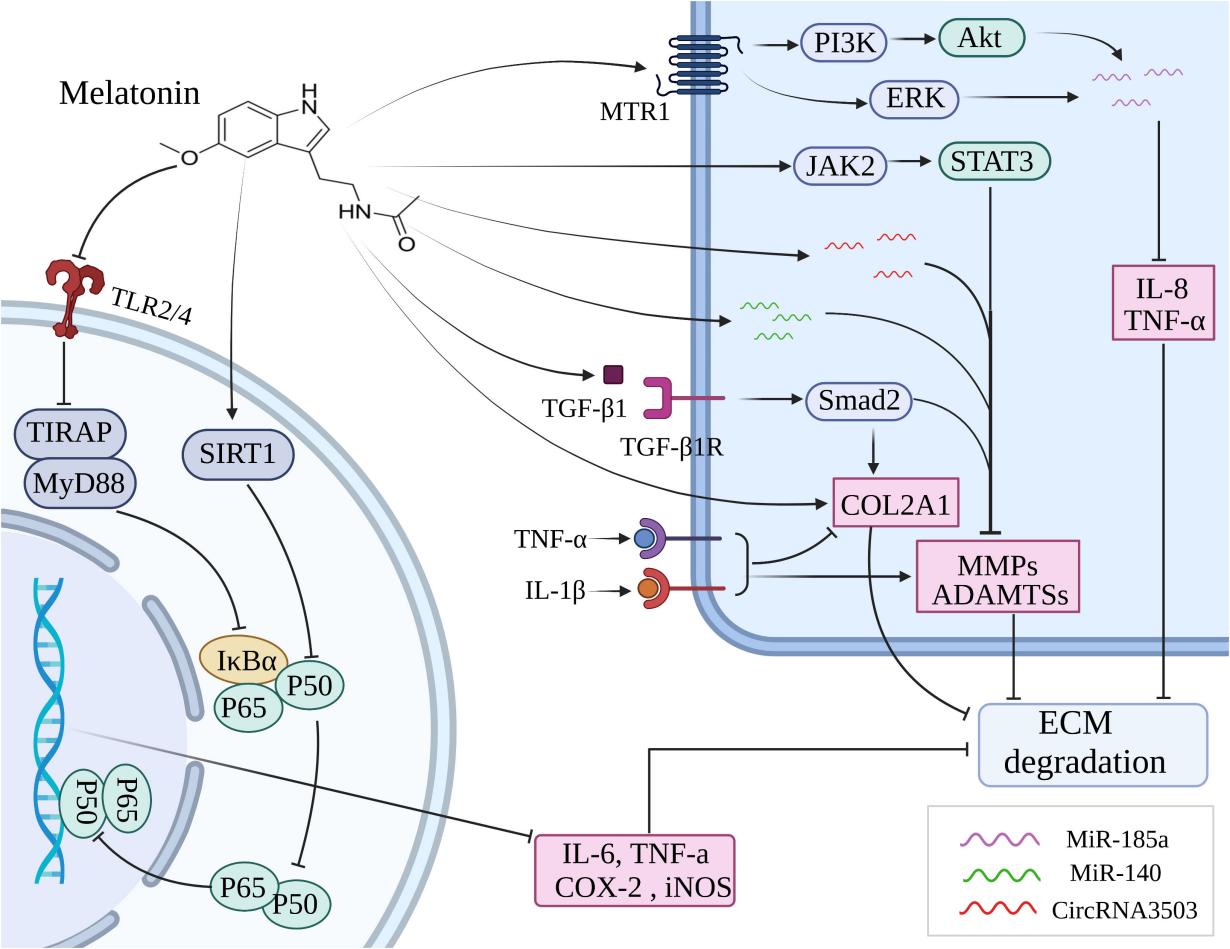
Melatonin inhibits inflammation through various signaling pathways to ameliorate the cartilage destruction in OA. ECM, extracellular matrix; MMP, matrix metalloprotease; ADAMTS, a disintegrin and metalloproteinase with thrombospondin motifs; MTR1, melatonin receptor 1; SIRT1, silent information regulator 1; TLR, toll-like receptor; COL2A1, collagen type II alpha-1.

The pathophysiology of OA is primarily influenced by chronic inflammation, as indicated by a growing body of research (156, 157). IL-1β is commonly utilized as an *in vitro* model to simulate the inflammatory process of OA. The treatment with IL-1β induced upregulation of MMP-3, MMP-9, MMP-13, ADAMTS-4, COX-2 and iNOS levels, while downregulation of chondrogenic marker COL2A1 in human mesenchymal stem cells (hMSCs) and chondrocytes. However, melatonin significantly mitigated the detrimental effects caused by IL-1β (154, 155, 158). By inhibiting the JAK2/STAT3 signaling pathway, melatonin effectively reduced the levels of MMP-3, MMP-9, and MMP-13, thereby attenuating cartilage degradation (138) (**Figure 3**). Moreover, Ke et al. have demonstrated that melatonin suppressed the production of IL-1β, IL-6, and COX-2 to mitigate the progression of OA in rats (159). It has been reported that TNF-a inhibited extracellular matrix synthesis by upregulating the expression of catabolic enzymes and downregulating the expression of anabolic enzymes in chondrocytes. Melatonin could effectively downregulate the levels of MMP-13 and upregulate the expression of COL2A1, thereby counteracting the inhibitory effect exerted by TNF-α on ECM (160) (**Figure 3**). Interestingly, this effect was further enhanced when combined with suitable exercise (160). The study conducted by Hong et al. demonstrated that the combination of melatonin and exercise treatment effectively suppressed abnormal catabolic upregulation, thereby reducing cartilage degradation (139). Besides, melatonin could directly bind to the MT1 receptor and thus inhibit the production of proinflammatory cytokines such as TNF-α and IL-8 in human OA synovial fibroblasts by antagonizing the PI3K/Akt and ERK signaling pathways, subsequently leading to an upregulation of miR-185a expression (140). It was found that melatonin enhanced the expression of miR-140, thereby abolishing IL-1β-induced matrix degradation in chondrocytes (141). In addition, melatonin could induce the upregulation of circRNA3503 to counteract the ECM degradation induced by TNF-α and IL-1β (161).

The NF-κB pathway plays a crucial role in orchestrating the expression of multiple proinflammatory cytokines, including IL-6, TNF-a, COX-2, and iNOS (162). Melatonin inhibited the activation of NF-κB stimulated by H_2_O_2_ and also blocked the phosphorylation of upstream signaling pathways including JNK, p38 MAPK, ERK, PI3K, and Akt to improve the anti-inflammatory effects in chondrocytes (142). The inhibitory effects of melatonin on NF-κB and its upstream signaling pathways markedly are reversed by the downregulation of the SIRT1 level, in other words, the SIRT1 pathway participates in the cytoprotective and anti-inflammatory effects of melatonin via the inhibition of NF-κB signaling pathways on H_2_O_2_-induced articular cartilage destruction (142). Zhao et al. likewise testified that melatonin downregulates IL-1β-induced phosphorylation levels of P65 and IκBα in chondrocytes via SIRT1 pathways, thus abolishing NF-κB activation to function in cytoprotective and anti-inflammatory effects (155). It has been demonstrated that the toll-like receptor (TLR) mediates inflammatory responses triggered by chemical and physical stressors, such as cytokines and mechanical damage, ultimately leading to the development of OA (163). Hence, targeting the TLR signaling pathway may potentially serve as an efficacious therapeutic strategy for OA by attenuating the inflammatory damage. It was shown that melatonin exerted its protective effect on chondrocytes against inflammatory damage by inhibiting the TLR2/4-MyD88-NF-κB signaling pathway (143).

The expression of nicotinamide phosphoribosyltransferase (NAMPT), the rate-limiting enzyme in NAD+ biosynthesis, is enhanced by SIRT1 (164, 165). Activation of SIRT1 also promotes the synthesis of nuclear factor of activated T cells 5 (NFAT5), thereby enhancing the expression of pro-inflammatory cytokines in articular cartilage, including IL-1β, IL-6, TNF-α, COX-2, and iNOS (166, 167). Guo et al. demonstrated that melatonin significantly alleviated the expression of MMP-3 and MMP-13 induced by IL-1β in chondrocytes through the inhibition of SIRT1-mediated NAMPT and NFAT5 signaling pathways (154). Moreover, melatonin enhanced the expression of COL2A1 by regulating SIRT1, thereby restoring dexamethasone-induced ECM deterioration in chondrocytes (168). Several studies have shown that the synthesis of ECM and the differentiation, migration, and adhesion of chondrocytes were all significantly influenced by TGF-β1 (169, 170). Activation of the TGF-β1/Smad2 pathway stimulated by melatonin in IL-1β-induced chondrocytes was found contributing to the synthesis of ECM (155). It was suggested that melatonin administration in chondrocytes increased the upregulation of key chondrogenic marker genes, including Sox9, aggrecan, and collagen II via the TGF-β1 signaling pathway (171).

### 3.4 Melatonin as a modulator of chondrocyte death

**Figure 4:**
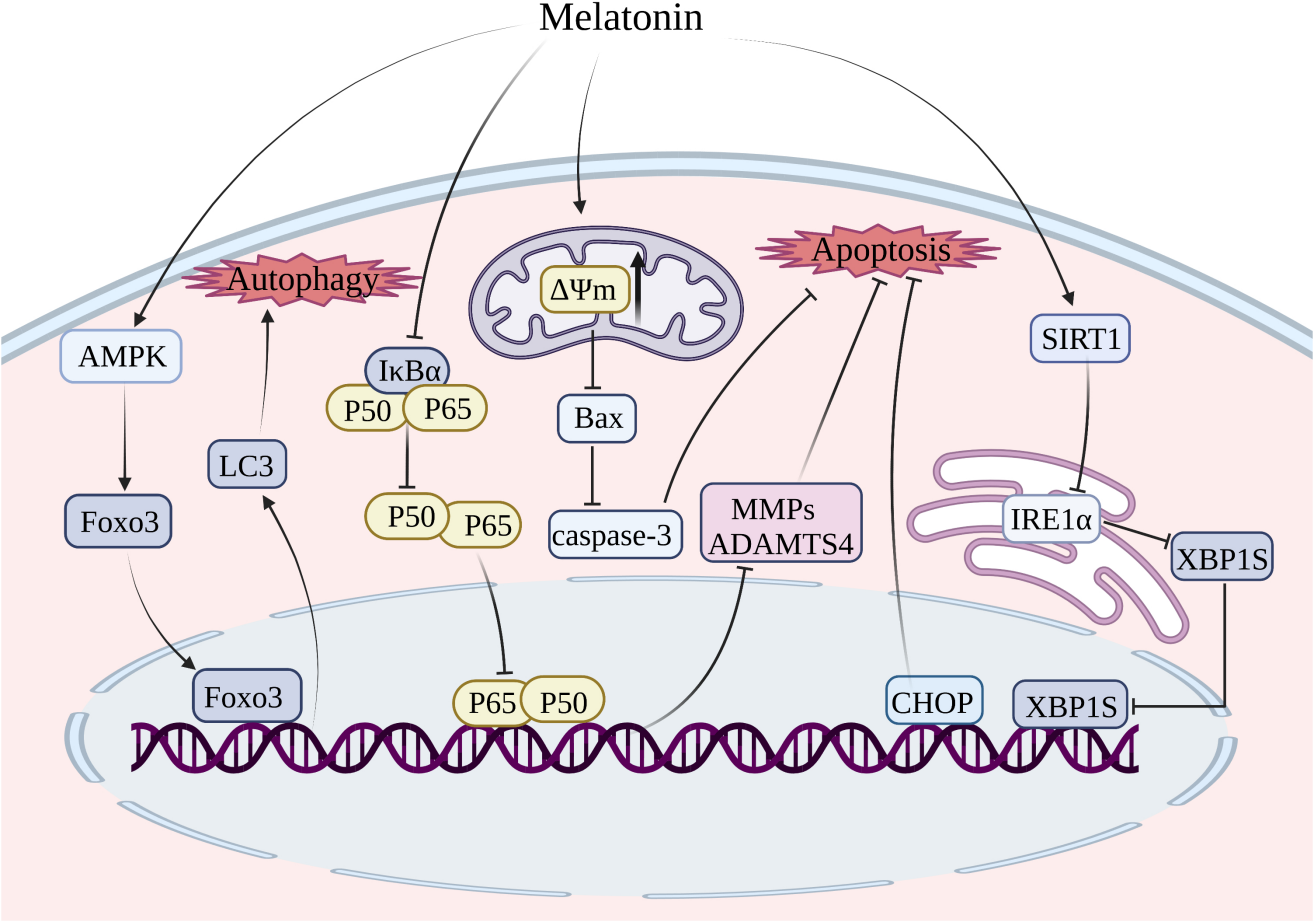
Treatment of melatonin inhibits chondrocyte apoptosis and autophagy. MMP, matrix metalloprotease; ADAMTS, a disintegrin and metalloproteinase with thrombospondin motifs; SIRT1, silent information regulator 1; Foxo3, forked box transcription factor Class O 3; IRE1-α, inositol-requiring enzyme 1-α; XBP1, X-box binding protein 1; CHOP, C/EBP homologous protein.

As the sole cell type in cartilage, chondrocytes function as the core factor in regulating the homeostasis in cartilage metabolism (172). Previous studies have indicated that chondrocyte apoptosis plays a significant role in the development of OA (173, 174). The initiation of apoptosis is believed to occur as an early response to the depolarization of mitochondria, which impairs the mitochondrial membrane’s potential. Substantial reductions in membrane potential promote permeabilization of the outer mitochondrial membrane, facilitating the release of apoptosis-related factors that trigger apoptosis (175, 176). Treatment with melatonin could restore the reduction of mitochondrial membrane potential and decrease the levels of caspase-3 and PARP, thereby ameliorating apoptosis in chondrocytes exposed to H_2_O_2_ (144) (**Figure 4**). A key regulator of energy homeostasis, known as 5’-AMP-activated protein kinase (AMPK), is a serine/threonine kinase composed of multiple catalytic subunits (α, β, and γ) (177). Multiple studies indicate that AMPK activation effectively inhibits apoptosis induced by the mitochondrial pathway through sustaining redox status and maintaining mitochondrial membrane potential, thereby restoring optimal mitochondrial function (178). The mammalian forked box transcription factor Class O (Foxo) family includes Foxo3, which functions as a downstream transcriptional factor in the AMPK signaling pathway and plays a crucial role in regulating antioxidant defenses and the autophagy process (179–181). Through the activation of AMPK/Foxo3 signaling pathways, melatonin exerted an inhibitory effect on apoptosis and induced upregulation of autophagy in chondrocytes to attenuate the progression of OA (144) (**Figure 4**).

In contrast to the internal mitochondrial pathway and extrinsic death receptor pathway, the ERS-mediated apoptosis pathway is initiated by the accumulation of misfolded proteins in the ER lumen, leading to ERS. The unfolded protein response (UPR) is a defensive mechanism that alleviates ERS and restores ER homeostasis (182, 183). However, if the ERS surpasses the threshold of UPR, it can trigger cellular apoptosis (184). UPR is initiated by transmembrane proteins, namely inositol-requiring enzyme 1-alpha (IRE1-alpha), protein kinase R-like ER kinase (PERK), and activating transcription factor 6 (ATF6) (185). The three primary signaling pathways in ERS-mediated apoptosis are IRE1α-X-box binding protein 1 (XBP1)-C/EBP homologous protein (CHOP), PERK-eukaryotic initiation factor 2α (eIF2α)-CHOP, and ATF6-XBP1-CHOP (186). The signaling pathway of IRE1α-XBP1-CHOP in chondrocyte apoptosis has been extensively investigated (187). The inhibition of the IRE1α-XBP1-CHOP signaling pathway is considered a promising target for delaying the progression of OA by blocking chondrocyte apoptosis (187). It was shown that melatonin enhanced the expression of SIRT1, which suppressed the IRE1α-XBP1-CHOP signaling pathway, thereby attenuating ERS-induced apoptosis in chondrocytes (145).

The utilization of BMSCs presents a promising strategy for alleviating articular cartilage degradation, given the wide availability of resources for harvesting BMSCs and their capacity to differentiate into various cell lineages including chondrocytes and osteoblasts (188, 189). The potential of regenerating damaged articular cartilage through the chondrogenesis of BMSCs is appealing, however, the inflammatory environment in cases of OA poses challenges for the survival of BMSCs. It has been demonstrated that the administration of melatonin could reduce the expression of Bax in IL-1β-induced BMSCs, thereby conferring protection to BMSCs against IL-1β-triggered apoptosis (190). Moreover, melatonin was found to inhibit the expression of proapoptotic markers such as ADAMTS4, MMP9, and MMP13, thus rescuing IL-1β-induced apoptosis of BMSCs and impaired chondrogenesis through the NF-κB signaling pathway (158).

As the leading risk factor for the development of OA, aging can induce senescence-associated phenotypes in joints, such as increased levels of cytokines, MMPs, and ROS, and reduced expression of aggrecan and collagen II (191). Due to its buffering and lubricating properties, hyaluronic acid plays a significant protective role in mitigating mechanical stresses on articular cartilage, and its synthesis can be hindered by chondrocyte senescence and death. It was reported that melatonin could effectively downregulate the expression of senescence-related proteins p16, p21, and p-p65, and thus counteract chondrocyte senescence and the subsequent downregulation of hyaluronic acid triggered by D-galactose through activation of the SIRT1 signaling pathway (192). Ferroptosis and pyroptosis, as novel forms of PCD, have been found implicated in the pathogenesis of OA (80, 118). Although there is currently no research reporting the impact of melatonin on ferroptosis and pyroptosis of chondrocytes, the antioxidative properties and anti-inflammatory actions of melatonin suggest its potential role as a significant inhibitor of ferroptosis and pyroptosis in chondrocytes.

## 4 Melatonin as a desirable pain-relieving drug in OA

OA is a primary contributor to chronic pain, significantly impacting the quality of life in individuals with OA. The exacerbation of chronic pain leads to sleep disorders, including reduced sleep efficiency and shortened total sleeping duration (193). The development of drugs to enhance the management of chronic pain in OA patients is therefore of utmost urgency. Numerous studies have suggested that melatonin exhibits analgesic effects in animal models of both acute and neuropathic pain (194–197). Several clinical trials have also confirmed the analgesic effect of melatonin in chronic pain conditions such as fibromyalgia, migraine headaches, and irritable bowel syndrome (198–200). Liu et al. demonstrated that the combination of melatonin and MT2 receptor yielded analgesic effects in rats with temporomandibular OA (201). The application of auricular acupressure has been found to enhance melatonin levels, thereby providing relief for chronic pain and addressing sleep disorders in elderly individuals with OA (202). The conventional therapeutic approaches for alleviating chronic pain, such as intra-articular steroid injections and oral nonsteroidal anti-inflammatory drugs (NSAIDs), are associated with undesirable side effects. For instance, long-term oral administration of NSAIDs can lead to gastritis and peptic ulcers, while repeated intra-articular steroid injections may result in decreased bone density and infection (203, 204). Significantly, melatonin to organs such as the liver and kidneys is associated with almost no toxicity and adverse effects (205). Collectively, the antioxidative, anti-inflammatory, and analgesic properties of melatonin render it a promising pharmaceutical agent for the treatment of OA.

## 5 Novel potential delivery systems of melatonin

**Figure 5:**
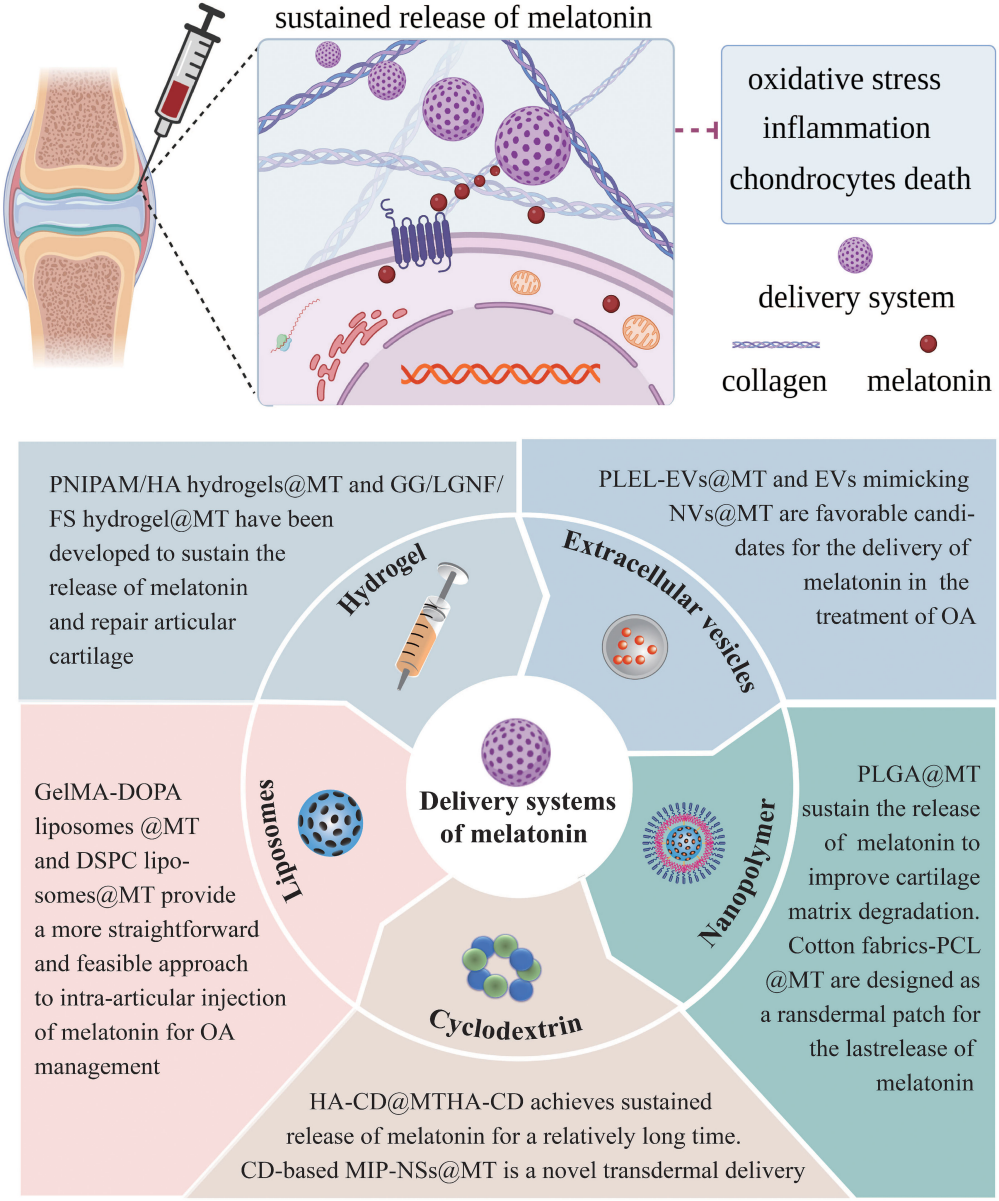
Melatonin sustained release delivery systems for the treatment of OA. MT, melatonin; PLEL, poly (D, L-lactide)-poly (ethylene glycol)-poly (D, L-lactide); EVs, extracellular vesicles; NVs, nanovesicles; PLGA, polylactic-co-glycolic acid; PCL, polycaprolactone; HA, hyaluronic acid; CD, cyclodextrins; MIP-NSs, molecularly imprinted nanosponges; GelMA-DOPA, gelatin methacryloyl-dopamine; DSPC, phosphatidylcholine; PNIPAM, poly (N-isopropyl acrylamide); GG, gellan gum; LGNF, lignocellulose nanofibrils; FS, forsterite.t of OA.

The closed nature of the knee joint and the absence of blood vessels in the articular cartilage pose challenges for medications to accumulate within the joint via systemic circulation, leading to reduced efficacy and potential systemic adverse effects. Intra-articular administration is considered the optimal method for treating joint disorders, as it allows direct delivery of the drug to the articular cavity, thereby overcoming the aforementioned disadvantage. The frequent intra-articular injections, however, are invasive procedures that incur additional expenses, diminish patient adherence, and increase the risk of infection (206). The development of novel drug delivery systems that minimize the frequency of injections may hold the key to overcome the limitations of conventional intra-articular injection, which lacks long-term efficacy. Due to the challenge of finding suitable delivery carriers to arrive at the chondrocytes, therapeutic or preventative options for healing damaged articular cartilage in OA remains limited (207). For this reason, numerous researchers have devoted themselves to developing melatonin sustained release delivery systems for the treatment of OA (**Figure 5**). Up to now, several promising melatonin sustained release delivery systems have been successfully developed (**Table 3**).

**Table 3:** The promising sustained release delivery systems of melatonin.

Materials	Delivery systems	Usage	Reference
Extracellular vesicles	PLEL-EVs@MT	injection	Tao et al. (161)
	EVs mimicking NVs@MT	injection	Kim et al. (208)
Nanopolymer	PLGA@MT	injection	Liang et al. (143)
	Cotton fabrics-PCL@MT	transdermal patch	Massella et al. (209)
Cyclodextrin	HA-CD@MT	injection	Zhang et al., 2022 (137)
	CD-based MIP-NSs@MT	transdermal patch	Hoti et al. (210)
Liposomes	GelMA-DOPA liposomes@MT	injection	Xiao et al. (211)
	DSPC liposomes@MT	injection	Ji et al. (212)
Hydrogel	PNIPAM/HA hydrogels@MT	injection	Atoufi et al. (213)
	GG/LGNF/ FS hydrogel@MT	injection	Kouhi et al. (214)

MT, melatonin; PLEL, poly(D, L-lactide)-poly(ethylene glycol)-poly(D, L-lactide); EVs, extracellular vesicles; NVs, nanovesicles; PLGA, polylactic-co-glycolic acid; PCL, polycaprolactone; HA, hyaluronic acid; CD, cyclodextrins; MIP-NSs, molecularly imprinted nanosponges; GelMA-DOPA, gelatin methacryloyl-dopamine; DSPC, phosphatidylcholine; PNIPAM, poly(N-isopropyl acrylamide); GG, gellan gum; LGNF, lignocellulose nanofibrils; FS, forsterite.

### 5.1 Extracellular vesicles

Extracellular vesicles (EVs), which can be classified into three subtypes based on their size, namely apoptotic bodies, ectosomes, and exosomes, are proteolipid nanoparticles secreted by diverse cell types including bacteria, archaea, and eukaryotic cells. Apoptotic bodies, ranging in diameter from 800 to 5,000 nm, are generated through cellular shedding during the process of apoptosis. Conversely, ectosomes are formed by the plasma membrane via budding mechanisms and have a size range of 50 to 1000 nm. Additionally, exosomes (40–200 nm) are secreted from intracellular multivesicular bodies that merge with the cytoplasmic membrane. Exosomes play a crucial role in facilitating intercellular communication by transporting lipids, proteins, and various nucleic acids such as mRNAs, circular RNA, and miRNA (215). Recent studies have demonstrated the significant potential of exosomes derived from MSCs as nano-carriers for delivering therapeutic genetic materials and drugs (216). Compared to MSCs, exosomes are non-viable, resulting in lower costs for storage and maintenance, as a viable state is needed in maintaining cells. Additionally, exosomes possess hypoimmunogenic properties and have a nano-scale size, which significantly reduce the likelihood of rejection (217, 218). Furthermore, exosomes possess the potential to traverse the blood-brain barrier, thereby facilitating the development of therapeutic interventions targeting the central nervous system (219). The versatility of exosomes allows for facile engineering to specifically target molecules. The current focus of numerous investigations lies in elucidating the mechanisms and functions of exosomes as efficacious drug delivery systems for various disorders. The ability of EVs to penetrate cartilage and target chondrocytes renders them as promising nano-carriers for therapeutic drugs in the treatment of OA (220). The evidence has demonstrated that EVs function as nano-carriers capable of delivering drugs to chondrocytes, thereby alleviating the progression of OA (161, 221). In conclusion, it is speculated that melatonin-loaded EVs can effectively penetrate articular cartilage and selectively target chondrocytes to attenuate the degeneration of articular cartilage by inhibiting oxidative stress, inflammation, and chondrocyte death.

The poly (D, L-lactide)-poly (ethylene glycol)-poly (D, L-lactide) (PDLLA-PEG-PDLLA; PLEL) triblock copolymer gels, which possess reversible, injectable, and thermosensitive properties, have been widely utilized in nano-drug delivery systems (222). The use of PLEL as a carrier for EVs has been employed to significantly enhance the sustained release of drugs loaded in EVs (161). The PLEL-entrapped EVs may offer promising delivery systems for achieving sustained release of melatonin, thereby presenting a potential therapeutic approach to alleviate the progression of OA. The limited production efficiency and laborious extraction and purification procedures of EVs, however, hinder the potential utilization of EVs in clinical practice. To address the challenges associated with EVs, EVs mimicking nanovesicles (NVs) which have similar biophysical characteristics to EVs have been generated via durably extruding cells through a microfilter (223, 224). EVs mimicking NVs are promising carriers which can be engineered to load with a variety of therapeutic drugs (225). Findings have demonstrated that melatonin-loaded EVs mimicking NVs effectively alleviated atopic dermatitis induced by 2,4-Dinitrofluorobenzene through the suppression of mast cell infiltration and local inflammation. Additionally, these EVs also promoted myocardial repair in cases of myocardial infarction by enhancing mitochondrial functions and reducing oxidative stress (208, 226). Taking together, it is very likely that the use of EVs mimicking NVs is a promising approach for delivering melatonin in the treatment of OA.

### 5.2 Nanopolymers

Nanopolymers possess exceptional mechanical properties, facile assembly, high biocompatibility, remarkable stability, scalability, and chemical modifiability, thereby offering favorable conditions for the design of nano-carrier delivery systems. Nanopolymers are broadly applied for the design of sustained release and site-specific drug delivery, resulting in improved therapeutic efficacy with fewer side effects (206, 227). Polylactic-co-glycolic acid (PLGA), a type of nanopolymer material, is widely employed in the field of drug delivery (228). The melatonin-loaded nano-delivery system was formed by encapsulating melatonin in PLGA, and the surface of PLGA was then modified with collagen II targeting polypeptides to enhance the targeting of such nanoparticles (143). What’s exciting was that the sustained release of melatonin for at least 14 days in the mice joint cavity was achieved by this nanoparticle, significantly reducing the frequency of injections compared to using melatonin alone (143). The PLGA nanoparticles loaded with melatonin enable precise targeting of cartilage and sustained release, thereby reducing the degradation of cartilage and the progression of OA. Consequently, intra-articular injection of these nanoparticles may represent a novel therapeutic approach for OA treatment.

Cotton fabrics functionalized by polycaprolactone (PCL) nanoparticles are designed as a transdermal patch for the release of melatonin (209). The biodegradation process of PCL could last up to one year, making it a widely used delivery system for sustained drug release (229). Melatonin-loaded PCL nanoparticles, when distributed on cotton fibers, exhibited a controlled and sustained release of melatonin (209). This transdermal delivery system significantly enhanced the skin permeation and sustained release of melatonin through a non-invasive approach. In conclusion, it is speculated that transdermal delivery systems may hold great potential in the treatment of OA.

### 5.3 Cyclodextrins

Cyclodextrins (CDs), which are cyclic oligosaccharides derived from starch hydrolysis, possess internal hydrophobicity and external hydrophilicity (230). Due to their unique characteristics, CDs have the ability to form host-guest complexes with suitable molecules to improve their stability, bioavailability, solubility, and controlled release. The CDs-based drug delivery system has facilitated the sustained release of the medication for OA treatment (231). The CD was incorporated into the hyaluronic acid (HA) solution to construct the HA-CD drug delivery system. Subsequently, melatonin was integrated into the HA-CD-based drug delivery system. The HA-CD melatonin delivery system achieved sustained release of melatonin over an extended period, effectively repairing dysfunctional mitochondria in OA chondrocytes (137). Furthermore, CDs could undergo polymerization with the cross-linking agent citric acid, followed by the addition of melatonin as a template molecule to form CD-based molecularly imprinted nanosponges (MIP-NSs) (210). Alongside these, CD-based MIP-NSs were incorporated into cream formulations to enhance their direct applicability to the skin. These skin formulations offered an innovative transdermal delivery system that enhanced the permeation of the skin and improved the sustained release of melatonin (210). The transdermal delivery systems of CD-based MIP-NSs provided a more advanced approach for delivering melatonin into the skin compared to traditional methods, such as intra-articular injection, thereby avoiding undesirable effects.

### 5.4 Liposomes

Liposomes have been extensively employed as drug delivery systems due to their exceptional biocompatibility and proficient capacity to regulate drug release (232, 233). Due to its excellent biocompatibility and strong adhesive properties, gelatin methacryloyl-dopamine (GelMA-DOPA) is widely used in the field of bone tissue engineering (234). The liposomes loaded with melatonin were combined with a GelMA-DOPA solution to fabricate the melatonin delivery system. The GelMA-DOPA liposomes delivery system was advantageous for regulating the sustained release of melatonin (211). The GelMA-DOPA liposomes delivery system for melatonin, although its application is limited to osteoporosis therapy right now, holds significant potential for alleviating OA. In addition, phosphatidylcholine (DSPC) liposomes could be employed as highly effective lubricants to reduce friction, in addition to their role as drug delivery systems (235). It was reported that the utilization of DSPC liposomes as a carrier for glucosamine sulphate enabled the delivery of effective boundary lubrication at the outermost layer of the joint while also facilitating the controlled and sustained release of glucosamine sulphate (212). As a result, DSPC liposomes may offer a more direct and practical approach for the intra-articular administration of melatonin in OA treatment.

### 5.5 Hydrogel

Hydrogel, a type of polymeric material, is extensively investigated in tissue engineering due to its exceptional biocompatibility, predictable degradation rate, appropriate elasticity, porous structure, and resemblance to the ECM (236, 237). The thermosensitive injectable hydrogel, poly (N-isopropyl acrylamide) (PNIPAM), has gained significant attention due to its ability to be directly injected into the injured area and effectively fill irregular flaws (238). PNIPAM has minimal cell adhesiveness and bioactivity despite its significant ability to replicate the architecture of some tissues. Accordingly, the combination of *in situ* injectable hydrogels with cells and bioactive compounds has garnered significant attention in the field of bone/cartilage tissue regeneration (239). The lubricating polysaccharide hyaluronic acid facilitated cellular adhesion, migration, and proliferation, thereby decreasing syneresis and hydrogel shrinkage (240). Various studies have shown that surface modification of PLGA with chitosan-g-acrylic acid (PLGA-ACH) can enhance adaptability, mucoadhesive properties, and regulate drug release (241, 242). The addition of PLGA-ACH particles as crosslinkers to PNIPAM enhanced the mechanical properties of PNIPAM, resulting in a closer resemblance to natural cartilage tissue (213). Simultaneously, the PLGA core acted as a carrier for the sustained release of melatonin (213). A previous study has demonstrated the efficacy of melatonin as a delivery system for cartilage tissue engineering, wherein injectable PNIPAM/hyaluronic acid hydrogels containing PLGA-ACH nanoparticles were applicated (213).

The low immunogenicity, cost-effectiveness, and ease of handling make Gellan gum (GG), which is composed of glucose, rhamnose, and D-glucuronate residues, particularly attractive for drug delivery (243). The GG-based hydrogel is used in cartilage regeneration due to its appealing characteristics, including non-cytotoxicity, biocompatibility, mild processing conditions, and structural resemblance to native glycosaminoglycans (237, 243). However, similar to other biodegradable hydrogels, it lacks the necessary mechanical strength and bioactivity required for reinforcement through nanoscale additions. Lignocellulose nanofibrils (LGNF), characterized by their high modulus, reactive surfaces, and large aspect ratio, present an ideal material for enhancing both the mechanical and biological properties of polymeric composites (244). The porous nanoparticle form of forsterite (FS), a crystalline member of the olivine family composed of magnesia and silicon, has been investigated for its potential as a sustained drug delivery system (245). Accordingly, in order to enhance the mechanical properties of GG-based hydrogel, LGNF and FS nanoparticles were incorporated, and thus an injectable delivery system based on GG/LGNF/FS hydrogel has been developed for sustained release of melatonin and articular cartilage repair (214).

## 6 Conclusions and future directions

Increasing evidence suggests that inflammation, oxidative stress, and chondrocyte death are closely linked to the severity and progression of OA, rendering them potential targets for OA treatment. The present review provides a comprehensive overview of the role of melatonin in modulating inflammation, oxidative stress, and chondrocyte death to attenuate OA progression through the regulation of various signaling pathways including SIRT1, Nrf2, NF-κB, JAK2/STAT3, TGF-β1/Smad2, AMPK/Foxo3, IRE1α-XBP1-CHOP, PI3K/Akt and ERK. Obviously, numerous signaling pathways are implicated in the potential mechanism of melatonin in the treatment of OA. The primary source of ROS leading to chondrocyte death and exacerbating inflammatory reactions, thereby aggravating articular cartilage degradation, is mitochondrial dysfunction. The melatonin-based treatment restores impaired mitochondrial functions by recovering reductions in membrane potential and enhancing the synthesis of ATP, mtDNA, and respiratory chain factors to alleviate oxidative stress and chondrocyte death. Although melatonin is considered as an effective antioxidant for maintaining mitochondria, yet the current research in this field still remains insufficient. Therefore, future studies should aim to comprehensively and profoundly investigate the interplay between melatonin and signaling pathways on mitochondria in OA chondrocytes.

The efficacy of EVs as a well-researched carrier has been demonstrated in the administration of melatonin for various disorders. However, there is a lack of direct research to substantiate the efficacy of melatonin-loaded EVs in the treatment of OA. The efficacy of melatonin-loaded EVs will be validated in future studies. In addition, various innovative bioactive materials, including nanopolymers, cyclodextrins, liposomes, and hydrogels, have been developed to ensure sustained release of melatonin and target articular cartilage. The use of novel carriers for intra-articular injection can effectively reduce the injection frequency, thereby optimizing the therapeutic efficacy and bioavailability of melatonin in the treatment of OA. Therefore, these biomaterials play an indispensable role in advancing the potential clinical efficacy of melatonin. In summary, combined melatonin with multiple bioactive agents holds great promise as a strategy for OA treatment.

## Author contributions

ZX: Writing – original draft, Data curation, Methodology, Software, Visualization. GP: Data curation, Methodology, Formal analysis, Funding acquisition, Investigation, Validation, Writing – review & editing. JD: Formal analysis, Funding acquisition, Methodology, Writing – review & editing. ML: Writing – review & editing, Investigation, Project administration, Supervision. XN: Project administration, Supervision, Writing – review & editing, Methodology, Resources. YZ: Methodology, Project administration, Supervision, Writing – review & editing, Conceptualization, Formal analysis. HY: Conceptualization, Project administration, Writing – review & editing, Funding acquisition, Investigation, Resources. HS: Conceptualization, Funding acquisition, Investigation, Project administration, Writing – review & editing, Formal analysis, Methodology.
